# Integrative Analysis of MALT1 as a Potential Therapeutic Target for Prostate Cancer and its Immunological Role in Pan-Cancer

**DOI:** 10.3389/fmolb.2021.714906

**Published:** 2021-12-02

**Authors:** Haotian Tan, Yaqi Xie, Xuebao Zhang, Shuang Wu, Hongwei Zhao, Jitao Wu, Wenting Wang, Chunhua Lin

**Affiliations:** ^1^ Department of Urology, The Affiliated Yantai Yuhuangding Hospital of Qingdao University, Yantai, China; ^2^ Department of Urology, The Affiliated Yantai Yuhuangding Hospital of Binzhou Medical University, Yantai, China; ^3^ Department of Reproductive Medicine, The Affiliated Yantai Yuhuangding Hospital of Qingdao University, Yantai, China; ^4^ Central Laboratory, Yantai Yuhuangding Hospital, Yantai, China

**Keywords:** prostate cancer, pancancer, Malt1, bioinformatics analysis, therapeutic target

## Abstract

**Background:** Mucosa-associated lymphoma antigen 1 (MALT1) is an oncogene in subsets of diffuse large B cell lymphoma (DLBCL) and mucosa-associated lymphoid tissue type (MALT) lymphoma. However, the role of MALT1 across cancers, especially in prostate cancer is still poorly understood.

**Methods:** Here, we used several public datasets to evaluate MALT1 expression. Then, PCa cell lines and nude mice were used to investigate the cellular functions *in vitro* and *in vivo*. Microarray data were downloaded from The Cancer Genome Atlas and MALT1 was subjected to gene set enrichment analysis (GSEA) and Gene Ontology (GO) analysis to identify the biological functions and relevant pathways. Additionally, the correlations between MALT1 expression and mismatch repair (MMR) gene mutation, immune checkpoint gene expression, tumor mutational burden (TMB), and microsatellite instability (MSI) were investigated by Pearson correlation analysis. Moreover, the correlation between MALT1 expression and tumor immune infiltration was analyzed by the Tumor Immune Evaluation Resource (TIMER) database.

**Results:** MALT1 overexpression was significantly correlated with MMR gene mutation levels and crucially promoted proliferation and colony genesis while reducing PCa cell apoptosis levels *in vivo* and *in vitro*. MALT1 expression showed strong correlations with immune checkpoint genes, TMB, and MSI in most cancers. The GO analysis indicated that MALT1-coexpressed genes were involved in heterotypic cell-cell adhesion, actin filament-based movement regulation, and action potential regulation. GSEA revealed that MALT1 expression was associated with several signaling pathways, including the NF-κB signaling, Wnt/β-catenin and TGF-β signaling pathways, in PCa. Additionally, MALT1 expression was significantly correlated with the infiltration of immune cells, including B cells, CD8^+^ T cells, dendritic cells and macrophages, and negatively correlated with CD4^+^ cell infiltration in PCa.

**Conclusion:** MALT1 expression is higher in pancancer samples than in normal tissues. MALT1 promoted proliferation and colony genesis while reducing PCa cell apoptosis levels, and MALT1 suppression could inhibit xenograft tumor establishment in nude mice. Furthermore, MALT1 expression is closely related to the occurrence and development of multiple tumors in multiple ways. Therefore, MALT1 may be an emerging therapeutic target for a variety of cancers especially PCa.

## Introduction

Prostate cancer (PCa), also called prostate adenocarcinoma (PRAD), is the most common malignant cancer apart from lung cancer in males worldwide, and the second leading cause of male cancer-related death in worldwide (Gasnier and Parvizi, 2017; Bray et al., 2018). Most patients with PCa usually receive localized radical prostatectomy, radiation therapy, proton beam therapy, and cryosurgery after diagnosis (Shipley et al., 1995; Donnelly et al., 2010; Hayden et al., 2010). Androgen deprivation therapy (ADT) or castration therapy is considered the first-line therapy for patients with locally advanced cancer or recurrent or metastatic disease (Perlmutter and Lepor, 2007). However, fatal therapy-resistant disease eventually emerges in most PCa patients (Miller et al., 2017; Moreira et al., 2017). Therefore, there is an urgent need for a detailed investigation of the underlying mechanisms of PCa to discover novel molecular targets for gene therapy in order to thereby improve the prognosis of patients with PCa.

Mucosa-associated lymphoma antigen 1 (MALT1), is a paracaspase that belongs to the caspase family of proteases and has arginine-specific cysteine protease activity (Ruland et al., 2003). Together with coactivator-associated arginine methyltransferase 1 (CARMA1, also known as CARD11) and B cell lymphoma 10 (BCL10), MALT1 assembles the CARMA1/Bcl10/MALT1 (CBM) complex that bridges proximal antigen receptor signaling events to the IkB kinase (IKK) complex, causes the degradation of inhibitor of κB (IKK), activates the NF-κB pathway (Turvey et al., 2014). T cell receptor (TCR) stimulation leads to cleavage of Regnase-1 at R111 by Malt1/paracaspase, freeing T cells from Regnase-1-mediated suppression. In addition to its scaffolding role in NF-κB activation, MALT1 acts as a protease to enzymatically cleave and inactivate multiple substrates, including several negative regulators of canonical NF-κB signaling, such as TNFAIP3/A20 (Coornaert et al., 2008; McAllister-Lucas et al., 2011; Afonina et al., 2015). A tumor-promoting role of MALT1 has been found in a subset of diffuse large B cell lymphoma (DLBCL) and MALT lymphoma, indicating MALT1 as an attractive anticancer drug target (Ngo et al., 2006; Hailfinger et al., 2009; Nagel et al., 2012). MALT1 was also recently reported to be aberrantly expressed in solid tumors, including glioblastoma multiforme (Jacobs et al., 2020), especially in PCa(Wang et al., 2016; Ashcraft et al., 2020). However, the role of MALT1 in tumors, especially PCa, has not been extensively investigated.

To this end, this study aimed to explore the effects of downregulation of MALT1 expression on malignant biological behavior of PCa cells both *in vivo* and *in vitro* and to investigate their possible mechanism. Following additional in-depth bioinformatics analysis on the basis of a public database, the function and signaling pathways of MALT1 were elucidated.

## Materials and Methods

### The Expression of MALT1 in Public Databases

We performed a systematic analysis based on the TCGA(Blum et al., 2018) and Genotype-Tissue Expression (GTEx) databases (Lonsdale et al., 2013) to explore the expression levels in various types of cancers. To determine the expression level of MALT1 in different tissues, GTEx tissue expression was downloaded from the GTEx web portal. In addition, gene expression microarray datasets of MALT1 expression in PCa compared to that in normal prostate tissues were retrieved from the National Center for Biotechnology Information (NCBI) (GEO datasets GSE45016, GSE55945, GSE17906, GSE17951, GSE26910 and GSE3325), then use the RMA method for standardization and normalization, and use the combat algorithm to remove batch effects. After merging, there are a total of 237 samples of expression profile data, of which 121 are tumor samples and 116 are normal samples. Box-and-dot plots were generated using ggplot2 package in R. Two-sided Wilcoxon rank-sum test was used for statistical comparison between two groups.

### Cell Culture and Cell Transfection

The human prostate cancer cell lines LNCaP and PC-3 were acquired from Cobioer (Nanjing, CN). Both cell lines were grown in Roswell Park Memorial Institute-1640 medium (Thermo Fisher Scientific, NY, United States) with 10% (FBS; Biyuntian Company, Shanghai, CN), and 1% penicillin-streptomycin (Solarbio, Beijing, CN); 37°C and 5% carbon dioxide were the most suitable conditions for cell incubation. Cells were harvested in the logarithmic phase of growth for all experiments.

shRNA for MALT1 (shMALT1, target sequence 5′-CCA​TTC​ACA​TCC​TGG​TAA​T-3′) and control shRNA (shCtrl, target sequence 5′- TTC​TCC​GAA​CGT​GTC​ACG​T-3′) were from Genesil Biotechnology (Wuhan, CN). shMALT1 and shCtrl was cloned into a lentiviral vector (PLKO.1-C1). Cell transfection was performed with Lipofectamine 2000 (Invitrogen, Shanghai, CN) following the manufacturer’s protocol. Nonspecific shRNA was used as a negative control (NC). LNCaP was transfected with lentiviruses carrying shCtrl and shMALT1 respectively, and so was PC-3. KD cell lines was constructed by LNCaP and PC-3 which were transfected with lentiviruses carrying shMALT1.At 48 h after transfection PCa cells were transduced with lentivirus to knockdown MALT1. The culture medium was changed to normal medium 8–12 h after infection. Cells were observed 72 h post-infection with fluorescent microscope to ensure a positive infection rate of >70%. The selective silencing of MALT1 was identified by Western blot analysis.

### RNA Isolation and Quantitative Real-Time PCR

Total RNA was isolated from LNCap and PC-3 cells transfected with lentiviruses.by using TRIzol reagent (Tiangen Biotech, Beijing, CN) according to the manufacturer’s instructions. Reverse transcription was carried out using a reverse transcription kit (Thermo Fisher Scientific, NY, United States) according to the kit instructions. The obtained complementary DNA (cDNA) was applied for later testing. SYBR is a marker used to conduct quantitative reverse transcription PCR (qRT-PCR). The reaction conditions were 50°C for 2 min and 95°C for 10 min, followed by 40 cycles of 95°C for 30 s and 60°C for 30 s. The relative expression levels of genes were calculated using the 2−ΔΔCt method. GAPDH was used as an internal reference. We performed qRT-PCR experiments on a LightCycler®480 II (Roche Life Science, CA, United States), and SYBR Green Master Mix was purchased from Roche.

### Western Blot Analysis

Phosphate-buffered saline (PBS) was used to wash the cultured cells, and radioimmunoprecipitation assay (RIPA) buffer was added. Quantification of protein concentration was performed by BCA Protein Assay Kit (Beyotime, Shanghai, CN). Protein isolated from polyacrylamide gel according to the relative molecular mass was transferred to a polyvinylidene difluoride (PVDF) membrane (Millipore, MA, United States).

After 1 h of blocking with 5% blocking solution at room temperature, membranes were incubated at 4°C for 12 h with primary antibodies, and the secondary antibody was then added. Rabbit monoclonal antibody against MALT1 was from Abcam (ab33921, Cambridge, United Kingdom). After each incubation, the membrane was washed five times with PBS with 0.05% Tween-20 (Malinckrodt Baker, NJ, United States) (5 min per wash). Finally, the protein bands were visualized through a chemiluminescence detection system (Pierce, IL, United States).

### MTT Cell Viability Assay

Cell viability along with proliferation were measured by MTT assay. Cells were evenly introduced to 96-well plates at 2,000 cells per well and cultured under routine conditions. 10 μL of 5 mg/ml MTT (Sigma-Aldrich, Shanghai, CN) was added to each well at each time-point, followed by a 4-h incubation after which the culture medium was changed with 100 μL DMSO. After 2–5 min of agitation, the microplate reader (Thermo Fisher Scientific, NY, United States) was used to measure the optical densities at 490 nm wavelength (OD490). Growth curves based on OD490.

### Colony Formation Assay

LNCap and PC-3 cells infected with lentivirus for 24 h were seeded into six-well plates at a density of 500 cells per well; then cultured for 10–12 days to allow colony formation. Cells were fixed with 10% neutral buffered formalin solution and stained with 0.01% crystal violet solution (Beyotime, Shanghai, CN). The number of colonies was counted after full decolorization with PBS.

### Cell Apoptosis Assay

After transfection, LNCap and PC-3 cells were digested with 0.25% trypsin and transferred to a centrifuge tube. After that, samples were washed with D-Hanks buffer at 4°C, washed with 1× binding buffer and then resuspended in 300 μL of 1× binding buffer. Then, 5 μL of Annexin V-APC solution was added to the cells, followed by a 15-min incubation in the dark. Propidium iodide staining solution was added to the suspension, and the cells were analyzed with a flow cytometer to determine the percentage of apoptotic cells.

### Migration Assay

Transwell chambers (Corning, United States) were used to assess prostate cancer cell metastasis ability. The upper inserts of 24-well Transwell chambers were added, and 2 × 10^4^ cells were resuspended in 0.1 ml of serum-free medium. Then, 0.6 ml of medium with 30% FBS was added to the lower compartments as a chemoattractant. After 24 h of culture, the cells on the surface of the membrane were gently removed with cotton buds, fixed with 4% paraformaldehyde (Dalian Meilun Biotechnology, Dalian, CN) for 15 min, washed three times with PBS, fixed and stained with 0.1% crystal violet for 30 min. The stained cells were photographed under an inverted fluorescence microscope (magnification ×200; Olympus, TKY, JPN). Five visual fields were observed in each group.

### Cell Invasion Analysis

The invasive ability of the cells was detected by the Boyden chamber method. The cells were collected with trypsin and resuspended in 0.1% (W/V) bovine serum albumin in serum-free medium. Then, 200 ml of cell suspension (2 × 10^5^ cells) was inoculated into the upper cavity of the splint, and a polyethylene terephthalate (PET) membrane (pore diameter: 8.0 μm) (SPL Lifesciences, Pocheon, Kr) precoated with matrix gel (BD Biosciences, San Jose, CA) was implanted into the upper cavity of the splint. The bottom hole of the 24-well plate was filled with a cell medium containing 10% (v/v) FBS with or without isoplumbagin. Dimethyl sulfoxide or isoplumbagin were separately added to the superior lumen cell suspension. After 24 h of incubation, the cells migrating to the bottom side of the PET membrane were fixed with 4% paraformaldehyde and stained with crystal violet. Photos of the cells stained under the PET membrane were acquired with the microscope. The number of cells was counted by ImageJ, and the relative invasive ability was calculated by the number of cells per unit area.

### Subcutaneous Xenograft

Male nude mice (aged 4–6 weeks) were purchased from Shanghai SLAC Laboratory Animal Co. Ltd. (SH, CN). All 10 male nude mice were divided into a normal control (NC) group and a knockdown (KD) group, with 5 mice in each group. 1 × 10^7^ LNCaP lentivirus-infected cells carrying shCtrl (NC group) or shPNO1 (KD group) for 7 days were subcutaneously injected into the right arm pit of each mouse. The length (L) and width (W) of tumours were measured from day 7 post-inoculation and every 7 days until day 31 (tumour volume = 3.14/6 × L × W × W). All mice were then killed by injection of an overdose of 2% pentobarbital sodium followed by cervical vertebra dislocation, and tumours were excised and measured for volume and weight.

For tissue morphology evaluation, hematoxylin and eosin staining was performed on sections of the embedded samples. Immunohistochemistry (IHC) staining for MALT1 and Ki-67 was performed on sections from the xenograft tumors.

### Correlation Between MALT1 Expression and the Abundance of Immune Infiltrates

In the present study, we downloaded the infiltrating immune cell scores of 33 cancer types from the Tumor Immune Estimation Resource (TIMER) dataset (https://cistrome.shinyapps.io/timer/), which contains 10,897 samples from different cancer types included in the TCGA dataset. Spearman correlation analysis was utilized to investigate the correlation of MALT1 expression and the scores of these immune cells (B cells, CD4^+^ T cells, CD8^+^ T cells, neutrophils, macrophages and dendritic cells) in PCa patients. In addition, the correlation between MALT1 expression and immune checkpoint marker levels was evaluated *via* the “correlation” module in PCa.

### Correlation Between MALT1 Expression and Mismatch Repair Gene Mutation

Mismatch repair (MMR) genes play a crucial role in the intracellular MMR mechanism (Meiser et al., 2020). The loss of the function of a key gene in this mechanism may lead to DNA replication errors that cannot be repaired, which in turn leads to more somatic mutations. MLH1, MSH2, MSH6, PMS2, and EPCAM are five MMR genes, and their expression level in multiple cancers were obtained from the TCGA database. Correlation between MMR gene expression levels and MALT1 expression levels was analyzed using Spearman’s correlation method.

### Association Between MALT1 and Tumor Mutation Burden and Microsatellite Instability

TMB is defined as the number of mutations per megabyte of a database, which is used to reflect the number of mutations in tumor cells and is a quantitative biomarker (Krieger et al., 2020). TMB was calculated by the sum of the number of non-silent mutations in the TCGA samples. We downloaded the TMB data of 33 tumors from TCGA. Here, the TMB of each tumor sample was counted separately, and the correlation between MALT1 expression and TMB was summarized using the Spearman rank correlation coefficient by using “ggstatsplot” R package.

MSI is defined as the change in the length of a microsatellite DNA caused by the insertion or deletion of repetitive units in tumor tissue (Bonneville et al., 2017). We analyzed the correlation between the expression of MALT1 and MSI in 33 tumors using the Spearman method. MSI data were obtained from Bonneville et al. regarding pan-cancer MSI. In this study, we calculated the MANTIS scores for most of the tumor samples in the TCGA database by calculating the differences in the distribution of alleles at each microsatellite locus in the tumor-normal tissue paired samples and taking the average as the MSI score value for the tumor-normal tissue paired samples.

### Weighted Gene Coexpression Network Analysis Based on the Expression of MALT1

A total of 499 prostate cancer samples were obtained by downloading PCa expression profile data from the TCGA database. According to the median expression of MALT1, patients in TCGA with PCa were divided into the MALT1-high group and the MALT1-low group. The differentially expressed genes were screened between the MALT1-high group and the MALT1-low group (FC > 1.2 or FC < 5/6, *p* < 0.05), and the differentially expressed genes were obtained. Soft power 20 was set for network construction and module detection and the R package WGCNA was used to construct the gene co-expression.

### Construction of the Correlation Network and Gene Ontology Enrichment Analysis

GO analysis of the most highly correlated genes (cor >0.4) by using the clusterprofiler and GOplot package in R software V.3.5.2. Then, these correlated genes were used to generate a heatmap using the R package pheatmap after Spearman correlation analysis.

### Gene Set Enrichment Analysis

According to the median value of MALT1 expression, the samples were divided into two groups: the MALT1-high group and the MALT1-low group. For GSEA of single gene, the enriched background set was *hallmark: h.all.v7.1.symbols.gmt*. In order to analyze the biological function of MALT1 in prostate cancer, single GSEA was performed by using the ClusterProfiler and org.Hs.eg.db packages. Raw data of PCa were downloaded from TCGA database for the analysis of MALT1 expression between PCa patients and normal prostate tissues.

### Statistical Analysis

All experiments were repeated at least three times. The statistical analysis in this project was performed using GraphPad Prism 6.0 (version 6, California, United States). First, the F test was used to check the quality of variances. The data with F < 0.05 were subjected to two-tailed Welch’s t-test, and those with F > 0.05 were subjected to two-tailed Student’s t-test. Each group of data is represented by the mean ± SD. The p-value was used to indicate the statistical significance between the groups. When *p* < 0.05, the results were considered statistically significant.

## Results

### The Expression Level of MALT1 in Various Kinds of Tissues

**FIGURE 1:**
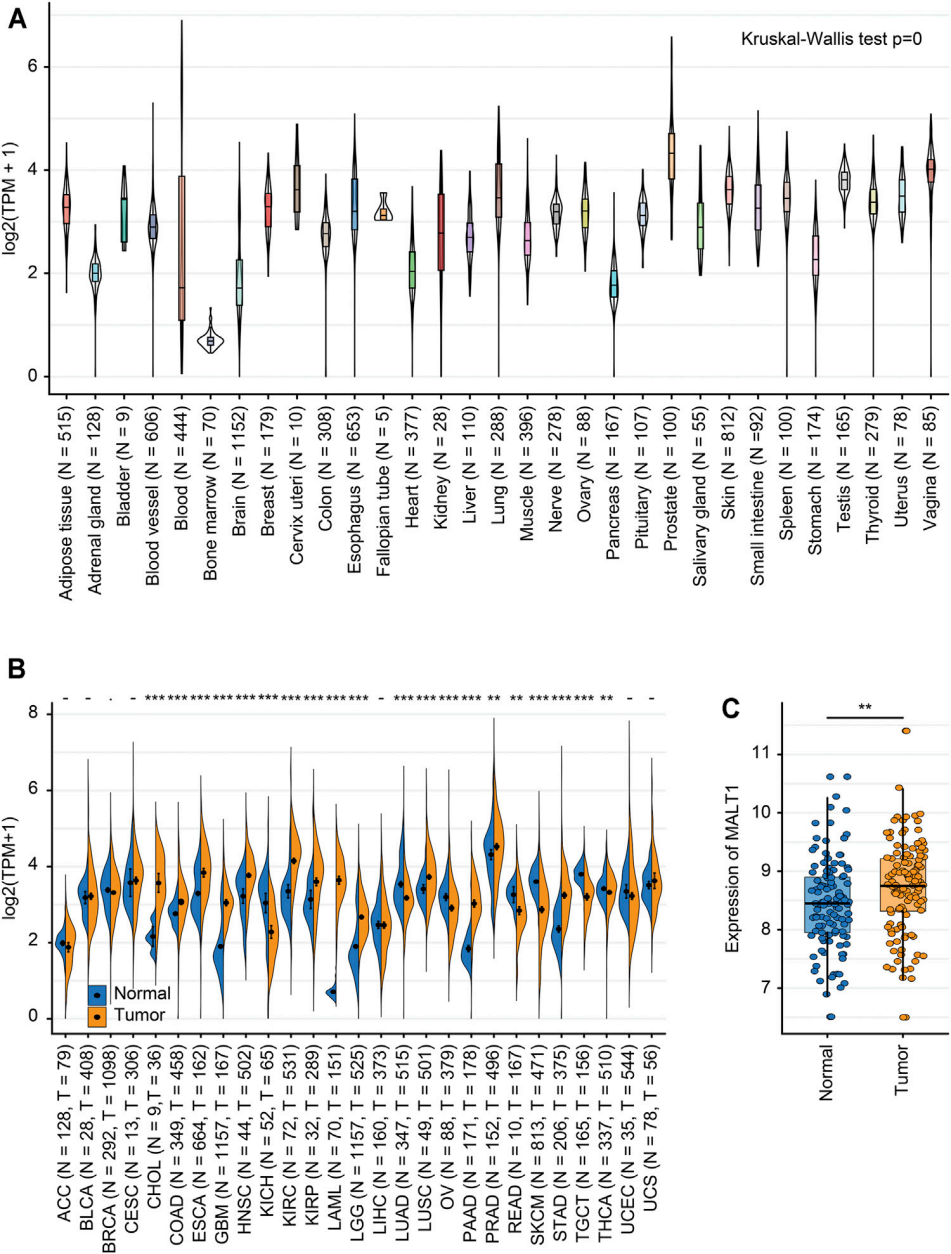
Expression levels of MALT1 in tissues from different origins and tumors. **(A)** Normal mRNA expression levels of MALT1 in different tissues from the GTEx database. **(B)** mRNA expression difference in MALT1 among normal, peritumor and tumor samples, combining data from the TCGA and GTEx databases. **(C)** mRNA expression difference in MALT1 between normal and tumor samples, based on data from the GEO database. * indicates *p* < 0.05, ** indicates *p* < 0.01, *** indicates *p* < 0.001.

We analyzed the MALT1 expression level in different tissues from the GTEx dataset, and there were significant differences among tissues (Figure 1A). Integration analysis of TCGA and GTEx RNA-seq data showed that compared to that in paired normal tissues, MALT1 was overexpressed in PRAD, cholangiocarcinoma (CHOL), colon adenocarcinoma (COAD), esophageal carcinoma (ESCA), glioblastoma multiforme (GBM), lung squamous cell carcinoma (LUSC), pancreatic adenocarcinoma (PAAD), and stomach adenocarcinoma (STAD) and was expressed at lower levels in kidney renal clear cell carcinoma (KIRC), lung adenocarcinoma (LUAD), ovarian serous cystadenocarcinoma (OV), skin cutaneous melanoma (SKCM), and rectum adenocarcinoma (READ) (Figure 1B). Using GEO databases to analyze MALT1 expression in the human PCa sample array also suggested that PCa tissues have significantly higher MALT1 expression than normal tissues (*p* < 0.005) (Figure 1C).

### Role of MALT1 in the Growth and Survival of PCa Cells

**FIGURE 2:**
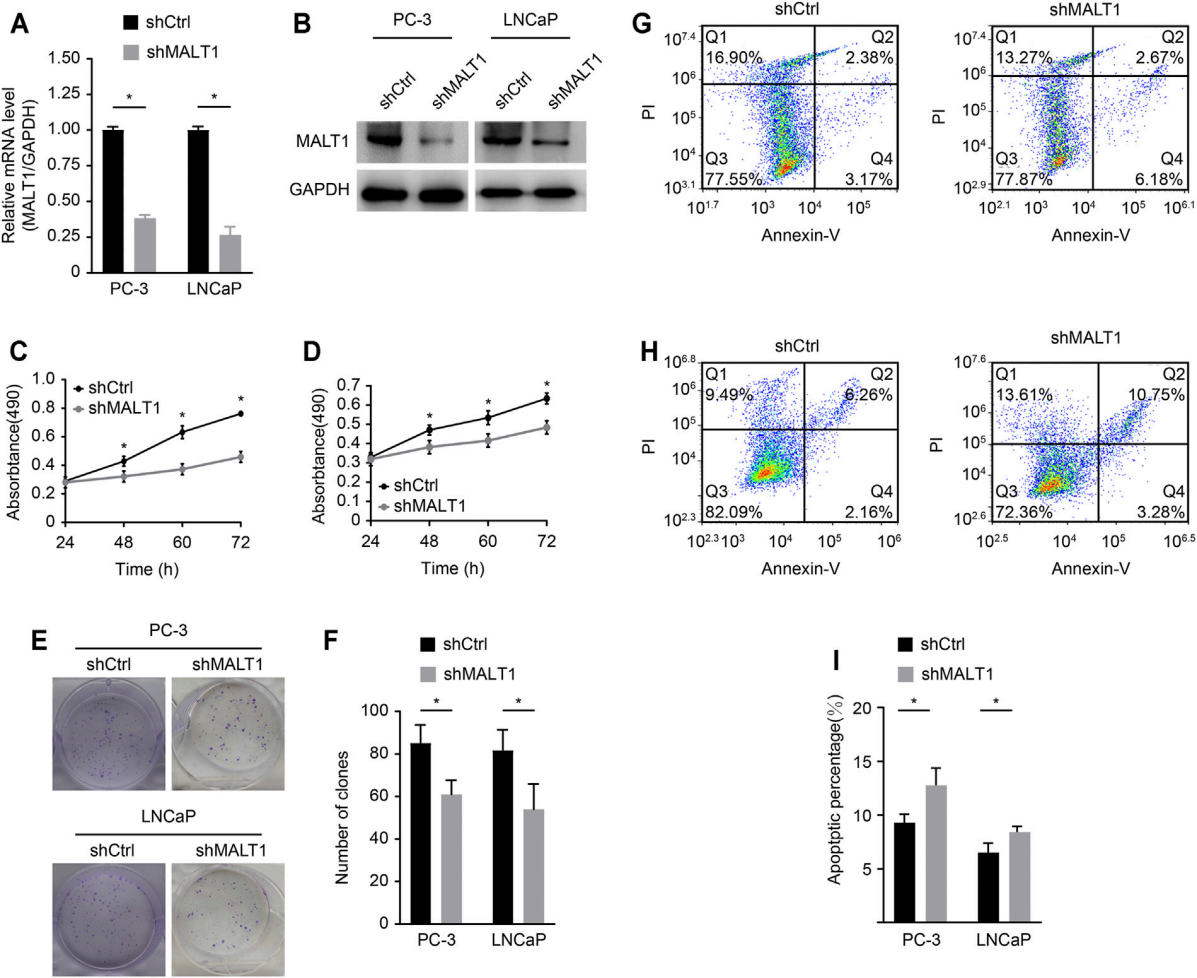
Role of MALT1 in the growth and survival of prostate cancer cells. **(A)** qRT-PCR results showing significantly reduced mRNA expression levels of MALT1 vs GAPDH in MALT1 KD cells. **(B)** Western blot showing reduced MALT1 protein expression in MALT1 KD cells. **(C)** Growth curves of PC-3 cells and **(D)** LNCaP cells measured by MTT assay. **(E, F)** Comparison of colony-forming ability between control and MALT1 KD cells. **(G)** The apoptosis ability of LNCaP cells and **(H)** PC-3 cells was detected by flow cytometry. Cells were divided into four quadrants: Q1: Annexin V-FITC -/PI +, which was representative of mechanical error; Q2: Annexin V-FITC+/PI+, which was representative of late apoptotic or necrotic cells; Q3: Annexin V-FITC -/PI-, which was representative of living cells; and Q4: Annexin V- FITC+/PI-, which was representative of early apoptotic cells. **(I)** Statistic analysis of **(G**, **H)**.

We studied the role of MALT1 in two PCa cell lines, PC-3 and LNCaP. MALT1 expression was then knocked down by MALT1-targeting shRNA (shMALT1) *via* lentivirus, and cells transduced with shCtrl were used as controls. The knockdown (KD) of MALT1 in these cell lines was confirmed by qRT-PCR, which showed 62% (*p* < 0.05) and 73% (*p* < 0.05) reductions in MALT1 mRNA levels in PC-3 and LNCaP cells, respectively (Figure 2A), and by Western blot (Figure 2B).

The effect of MALT1 KD on the proliferative activity of PC-3 and LNCaP cells was studied using the MTT assay (Figures 2B,C). The results showed that compared with that of the control cells (shCtrl), the proliferation of MALT1 KD cells (shMALT1) was significantly attenuated in both cell lines. It was also confirmed that for both cell lines in the KD group, the colony genesis ability was significantly decreased (*p* < 0.05 (keep the same as 0.05); Figures 2D,E), while cell apoptosis was significantly activated (*p* < 0.05; Figures 2F,G). These results indicated that MALT1 was important for the proliferation and survival of prostate cancer cells.

### Downregulation of MALT1 Expression Inhibits the Invasion and Migration of PCa Cells

**FIGURE 3:**
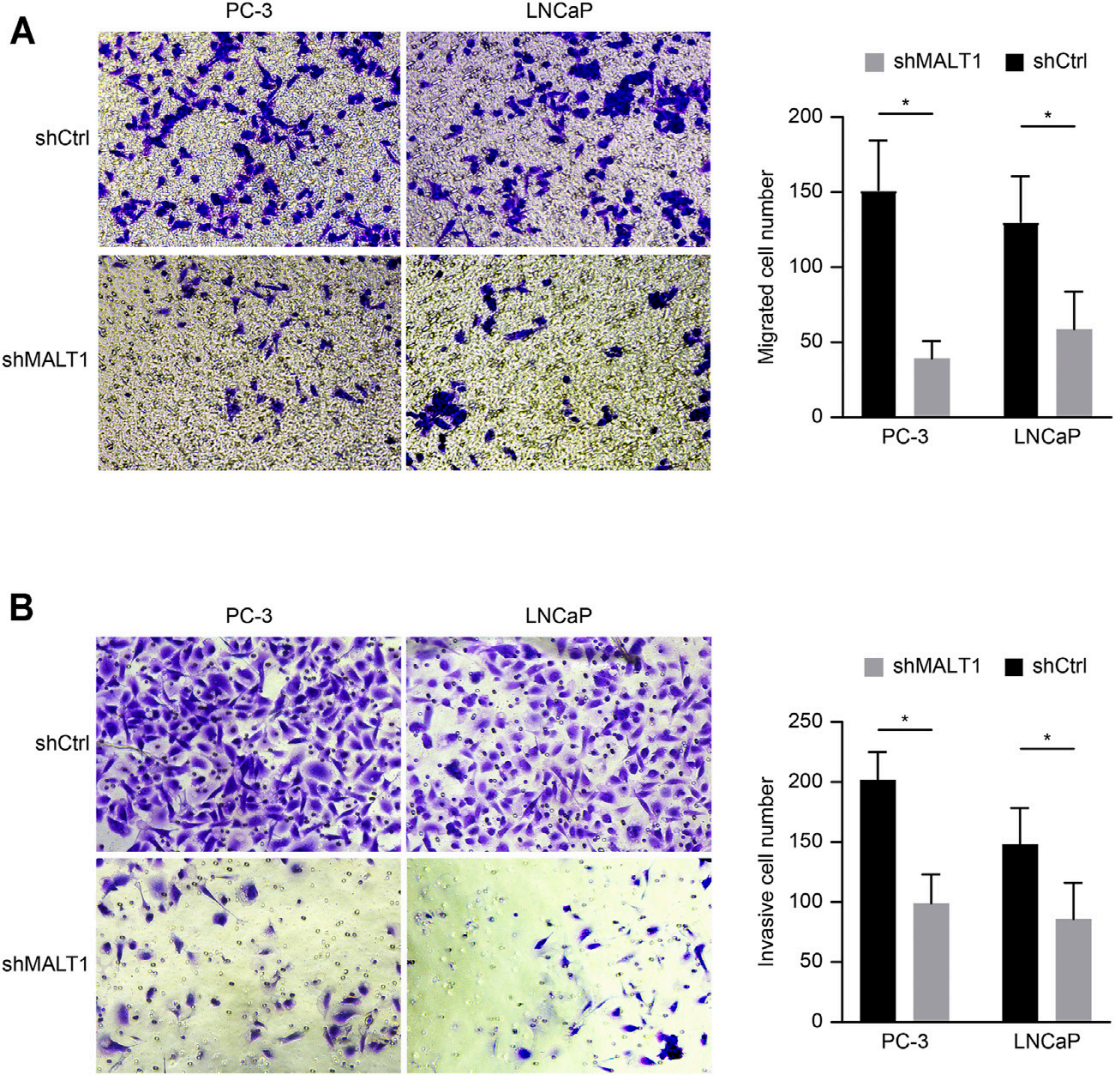
Downregulation of MALT1 inhibited the migration and invasion of PCa cells. PCa cells were treated with a lentiviral vector for 24 h. Then, **(A)** PCa cell migration ability was tested using a Transwell migration assay. **(B)** PCa cell invasion ability was tested using a Transwell invasion assay. Total magnification of all images is 200×. Data are shown as mean ± SD based on at least three independent experiments.

Transwell assay chambers with a Matrigel coating were used to evaluate tumor aggressiveness. The cells observed to degrade the Matrigel and pass through the membrane were counted, and the results revealed that the number of aggressive PC-3 cells was lower in the KD group than in the control group. Similar results were obtained for LNCaP cells (*p* < 0.05; Figure 3A). To evaluate whether downregulation of MALT1 expression affects the metastatic ability of PC-3 and LNCaP cells, we performed a Transwell experiment. The results indicated that MALT1 KD significantly reduced cell migration (*p* < 0.05; Figure 3B).

All these results demonstrated that after downregulating the expression of MALT1, the invasion and migration ability of tumor cells decreased, and the difference was statistically significant.

### Attenuation of Tumor Growth *in vivo* After MALT1 Knocking Down

**FIGURE 4:**
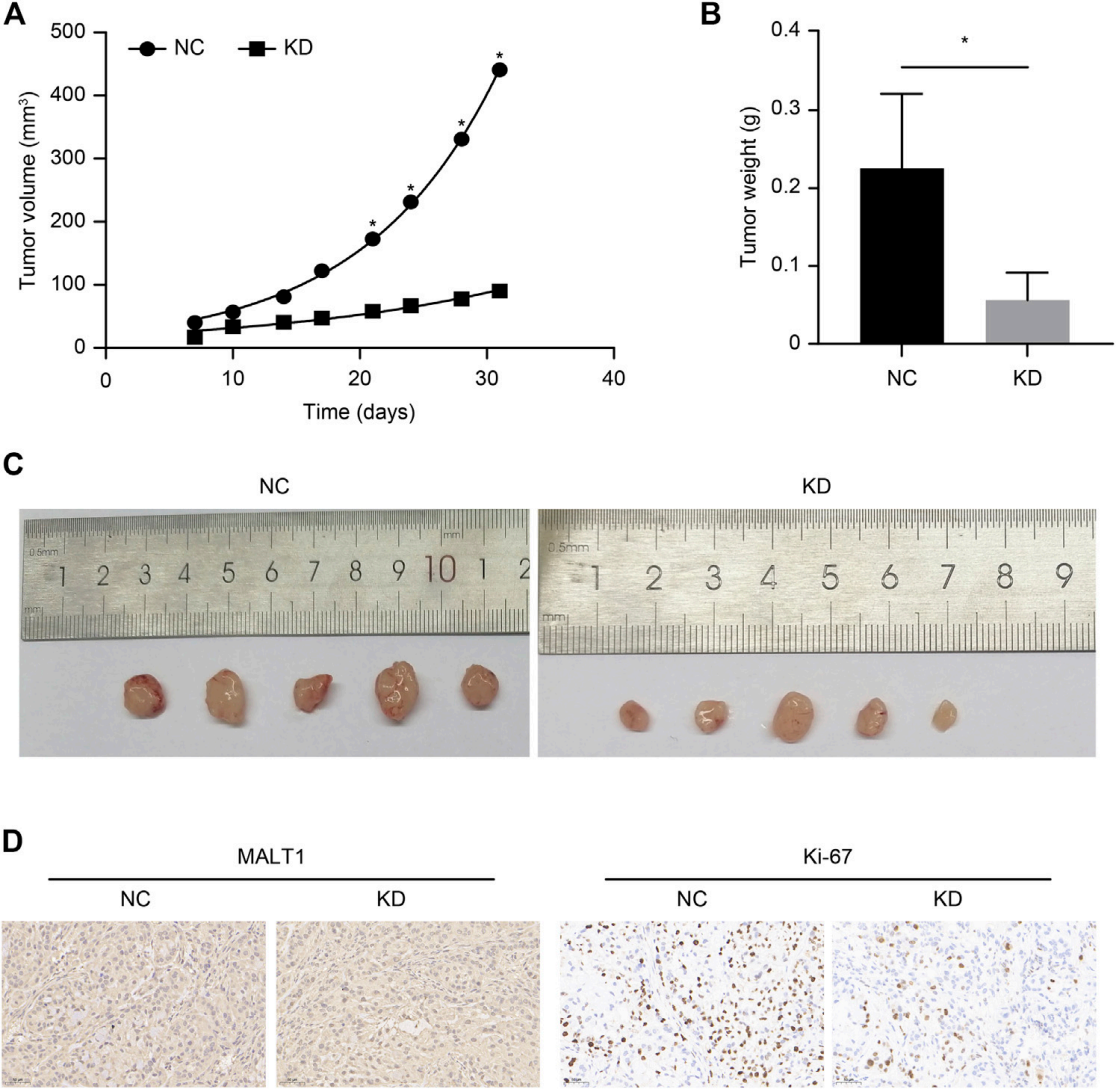
Subcutaneous xenotransplantation of PC-3 human PCa cells in BALB/c nude mice. Comparison of the volume of xenografts between the NC (shCtrl) and KD (shMALT1) groups of mice. **(A)** Comparison of the volume and **(B)** weight of xenografts on day 31 post-inoculation between the NC and KD groups of mice. **(C)** Representative photographs of the tumors. **(D)** IHC analysis showing a decrease in MALT1 and Ki-67 expression.

We established xenograft models to investigate the tumorigenic potential of MALT1 in prostate cancer; 10 male nude mice were subcutaneously inoculated with LNCaP cells expressing shCtrl (NC group) or shMALT1 (KD group), with 5 mice in each group. Tumor size was measured from the seventh day after inoculation (Figure 4A), and the mice were sacrificed on the 31st day. Tumor volume and tumor weight in the NC group were 440.93 ± 118.66 mm^3^ and 0.225 ± 0.096 g, respectively, which was compared with 90.04 ± 48.52 mm^3^ (*p* < 0.05) and 0.056 ± 0.035 g (*p* < 0.05) in the KD group (Figures 4B,C). Moreover, IHC analysis revealed that MALT1 and Ki-67 levels were substantially decreased in the KD group (Figure 4D). In consistent with *in vitro* results, the volumes and weights of tumors formed by MALT1 KD cells were significantly smaller and lighter tumors than those of tumors formed by control cells *in vivo*.

### WGCNA and GO Enrichment Analysis

**FIGURE 5:**
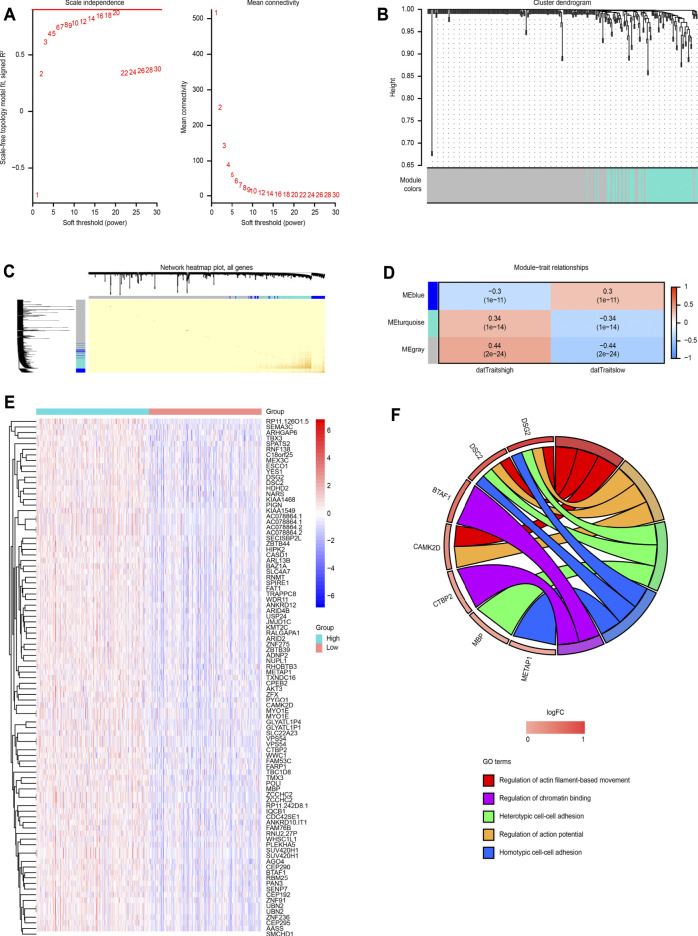
WGCNA based on MALT1 expression and GO analysis. **(A)** Screening out the soft-thresholding power through scale independence and mean connectivity. **(B)** Gene clustering tree (dendrogram) obtained by hierarchical clustering of adjacency-based dissimilarity. **(C)** Visualization of TOM of co-expressed genes in different modules by a heat map. Light colors indicate low overlap and dark red indicates high overlap. The darker color blocks along the diagonal are coexpression modules. **(D)** Module-trait relationship representing the relationship between modules and traits (high gene expression group, low gene expression group). The numbers inside each cell are the coefficient value and P value, and the color of each cell is assigned based on the correlation value of the module; red represents a strong positive correlation, and blue represents a strong negative correlation, The stronger the correlation, the darker is the color. **(E)** Heat map of MALT1 coexpressed genes each row and column represent one specific gene and patient, respectively; **(F)** GOplot representation of the analysis of the gene ontology (GO) items enriched among the coexpressed genes. The left side of the circle displays the gene, and the right side shows the GO item. The assorted colors represent different GO items, and the color of each GO item is annotated below the circle. If the gene belongs to a GO item, there will be a line between the gene and the GO item. The z-score (bottom) shows log2 (gene foldchange).

A total of 1,540 differentially expressed genes were screened between the MALT1-high group and the MALT1-low group (FC > 1.2 or FC < 5/6, *p* < 0.05). The expression profile was constructed for WGCNA and further analysis. WGCNA was performed with soft-thresholding power = 20 and obtained three modules, among which the turquoise module and blue module were significantly correlated with the traits of high and low expression of MALT1, respectively (Figures 5A–D).

A total of 86 coexpressed genes were obtained from the genes with a significant positive correlation with MALT1 expression (cor >0.4). A heat map of the coexpressed genes is shown in Figure 5E. GO functional enrichment analysis of the 86 coexpressed genes showed that they were related to heterotypic cell-cell adhesion, regulation of actin-based movement, regulation of action potentials and other functions, as shown in Figure 5F.

### Correlation Between the Expression of MALT1 and Immune Checkpoint Genes

**FIGURE 6:**
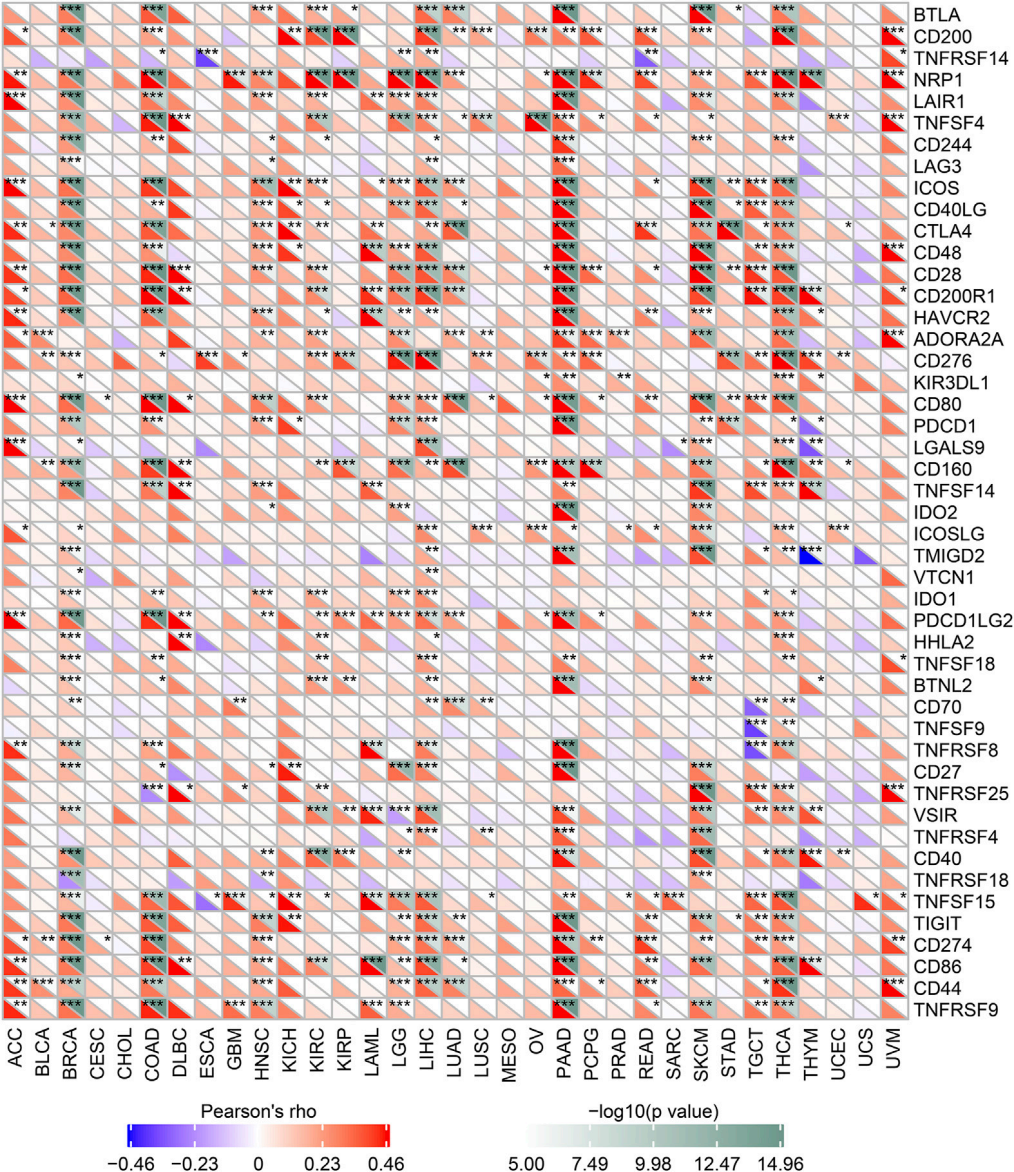
Correlation between MALT1 expression levels and acknowledged immune checkpoint genes expression in multiple tumors from the TCGA database. The correlation between of MALT1 expression level and immune checkpoint gene expression level in pan-cancer. The abscissa lists the cancer names and the ordinate lists the gene names. The lower triangle in each tile indicates coefficients calculated by Pearson’s correlation test, and the upper triangle indicates the log10-transformed P-value. *p < 0.05, **p < 0.01, ***p < 0.001.

We analyzed the correlation between MALT1 and immune checkpoint genes and found that MALT1 was associated with immune checkpoints in most tumors. In PAAD, breast invasive carcinoma (BRCA), and COAD, MALT1 expression was positively correlated with that of most immune checkpoint genes. In PRAD, expression of ADORA2A and KIR3DL was positively correlated with MALT1 expression, but that of other immune checkpoint genes was not (Figure 6).

### Correlation Between MALT1 Expression and Tumor Mutational Burden and Mismatch Repair Genes

We compared the immune infiltration levels among tumors with the presence of different somatic copy number alterations for the MALT1 gene by performing a TIMER analysis.

**FIGURE 7:**
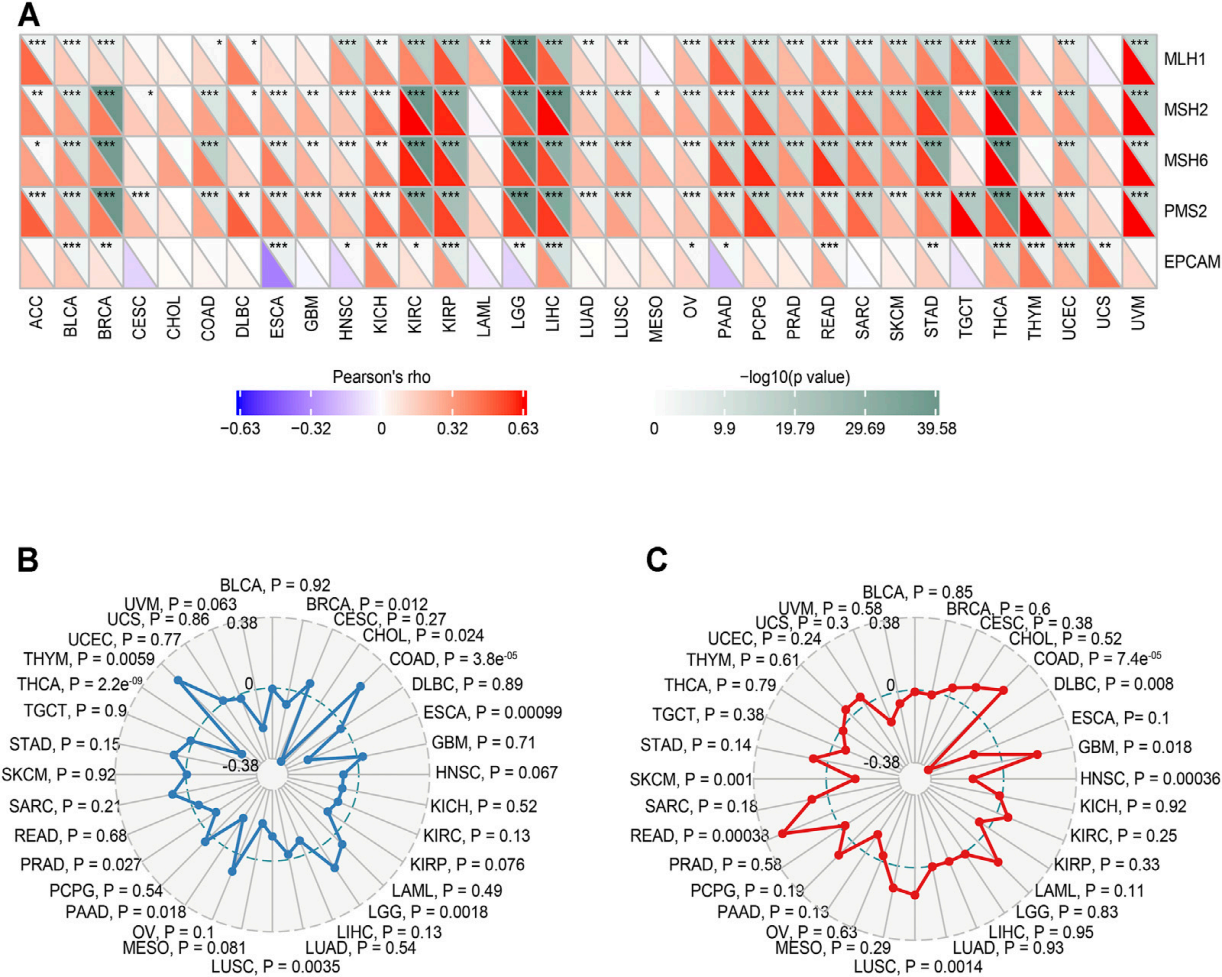
Relationship between MMR defects, TMB, MSI and MALT1 mRNA expression levels in various tumors in the TCGA database. **(A)** Correlation between MALT1 and five significant MMR genes (MLH1, MSH2, MSH6, PMS2, and EPCAM) in 33 types of cancers. The lower triangle in each tile indicates coefficients calculated by Pearson’s correlation test, and the upper triangle indicates the log10-transformed P-value. *: *p* < 0.05, **: *p* < 0.01, ***: *p* < 0.001. **(B)** Correlation between TMB and MALT1 expression, the black value is the scale of the correlation coefficient. **(C)** Correlation between MSI and MALT1 expression, the black value is the scale of the correlation coefficient. Spearman correlation test; *p* < 0.05 was considered significant.

We analyzed the correlation between MALT1 and MMR genes (MLH1, MSH2, MSH6, PMS2, and EPCAM). The results showed that there was a significant positive correlation between MLH1, MSH2, MSH6, and PMS2 and MATL1 in PRAD, liver hepatocellular carcinoma (LIHC), lower grade glioma (LGG), and thyroid carcinoma (THCA) (Figure 7A).

The relationship between MALT1 gene expression and TMB was analyzed by the Spearman correlation coefficient. The results showed that there was a significant negative correlation between MALT1 expression and TMB in PRAD, THCA, BRCA, CHOL, and ESCA (cor <0, *p* < 0.05) but a significant positive correlation between MALT1 expression and TMB in COAD, LGG, and thymoma (THYM) (cor >0, *p* < 0.05) (Figure 7B).

The correlation between gene expression and MSI was also analyzed by the Spearman correlation coefficient. The results showed that there was a significant negative correlation between MSI and MALT1 expression in SKCM, lymphoid neoplasm diffuse large B-cell lymphoma (DLBC), and head and neck squamous cell carcinoma (HNSC), while there was a significant negative correlation between MSI and MALT1 expression in COAD, GBM, LUSC, and READ (Figure 7C).

### Correlation of MALT1 Expression With Tumor-Infiltrating Immune Cells and GSEA

**FIGURE 8:**
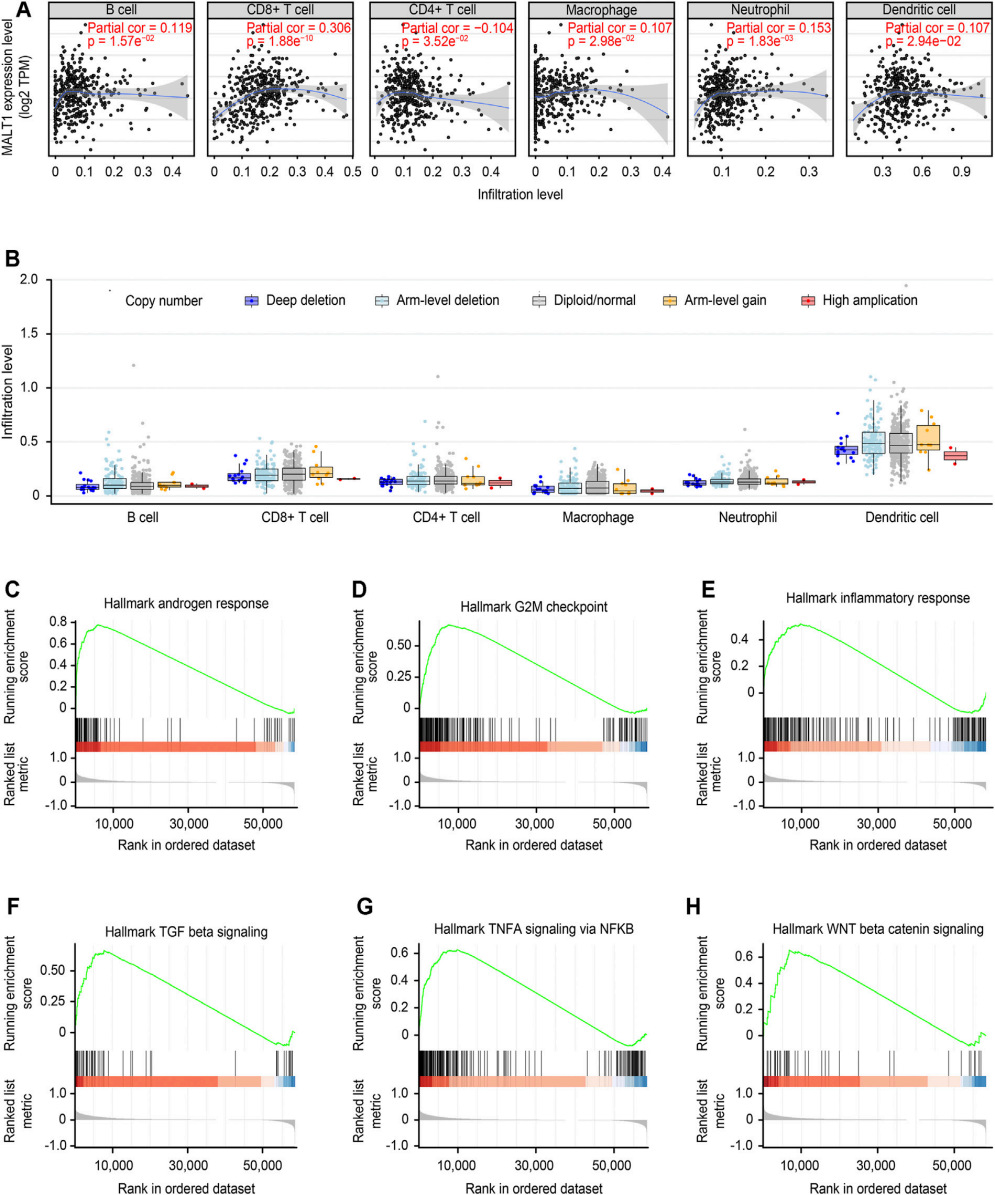
Immune correlation of MALT1 in PCa and GSEA of MALT1. **(A)** T Correlation of MALT1 expression level with immune infiltration level (B cells, CD4^+^ T cells, CD8^+^ T cells, neutrophils, macrophages, and dendritic cells) in PCa, as calculated by TIMER with data from the TCGA database. **(B)** Comparison of tumor infiltration levels among tumors with different somatic copy number alterations (SCNAs) for MATL1. SCNAs were defined by GISTIC 2.0, including deep deletion (−2), arm-level deletion (−1), diploid/normal (0), arm-level gain (1), and high amplification (2). Box plots are presented to show the distributions of each immune subset at each copy number status in selected cancer types. The infiltration level for each SCNA category was compared with the normal using a two-sided Wilcoxon rank-sum test; **(C–H)** Gene set enrichment analysis (GSEA) for the expression level of MALT1 and the signal pathways activated were present.

The correlation between MALT1 expression and tumor-infiltrating immune cells was analyzed by the TIMER database. The results showed that MALT1 expression was positively correlated with the infiltration of B cells, CD8^+^ cells, macrophages, and dendritic cells and negatively correlated with the infiltration of CD4^+^ cells. Among them, the correlation with CD8^+^ cells were the most significant (Figure 8A, Cor = 0.306, *p* < 0.05).

Furthermore, the correlation between the change in somatic cell copy number of the MALT1 gene and immune infiltration was analyzed. Copy number variation analysis showed that high amplification of MALT1 was correlated with CD8^+^ cell infiltration (*p* < 0.05), while deep deletion of MALT1 was associated with CD4^+^ cell infiltration (*p* < 0.05).

According to the median value of MALT1 expression, the samples were divided into a MALT1-high group and a MALT1-low group. GSEA showed that MALT1 was associated with several tumor-related pathways, including the androgen response, G2M checkpoint, NF-κB signaling pathway, Wnt/β-catenin pathway and TGF-β signaling pathway (Figures 8C–H).

## Discussion

PCa is a malignant disease that seriously endangers the health of men, and the molecular mechanism of its pathogenesis is very significant. Many studies have attempted to clarify hub genes that have an important impact on the development and metastasis of PCa at the transcriptome level (Varambally et al., 2005; Uhlén et al., 2015).

MALT1 is a well-known immune cell effector protein that manages adaptive immune responses driven by NF-κB (Jaworski et al., 2016). Enhanced expression of MALT1 has been linked to the development of leukemia and lymphoma (Xu et al., 2015; Saba et al., 2017). Therefore, MALT1 has been proposed to be a promising therapeutic target for the treatment of lymphomas and autoimmune disorders. MALT1 has been proven to be overexpressed in various solid tumors, and increasing numbers of research studies have confirmed that MALT1 is a key regulator of tumor development (Pan et al., 2016). According to Dai et al, MALT1 gene transcription tripled in pancreatic cancer cells cocultured with mouse dorsal root ganglion (Dai et al., 2007). Liu et al. revealed that MALT1 played an important role in cell proliferation *in vitro* and *in vivo* by inducing G1 phase arrest and confirmed that the activation of NF-κB could be significantly blocked by MALT1 inhibition and further regulates the proliferation and survival of glioblastoma pleomorphic cells (Liu et al., 2020).

According to the GEO, TCGA and GTEx datasets, the expression of MALT1 in PCa tissues was significantly higher than that in normal tissues and paracancerous tissues. Next, bioinformatics analysis of the functions and mechanisms of MALT1 was conducted. GO enrichment analysis with the genes positively related to MALT1 expression and GSEA revealed that MALT1 is related to heterotypic cell-cell adhesion, regulation of actin-based movement, and regulation of action potentials. Recent studies have indicated that the NF-κB pathway can be used as a target for castration-resistant PCa(McCall et al., 2012) and that the Wnt/β-catenin pathway is involved in the proliferation, invasion and metastasis of PCa (Clevers and Nusse, 2012; Zhang and Li, 2020). In addition, through GSEA, MALT1 was found to be associated with several cancer-related signaling pathways that promote cancer processes, including NF-κB pathway and Wnt/β-catenin pathway. All these above results support the protumor role of MALT1 in PCa.

Deregulation of cell proliferation and evasion of apoptosis are two hallmarks of cancer cells. In this study, knocking down MALT1 in PCa cell lines distinctly suppressed cells proliferation, migration, and invasion, and promoted apoptosis *in vitro*. In addition, the results of *in vivo* assays confirmed the tumor-promotive roles of MAL1 in PCa growth. Through the experiments reported here, we confirmed that MALT1 acts as a proto-oncogene and plays an important role in the development of PCa.

The TME has been a recent focus of tumor research. Since the tumor microenvironment was composed of a large number of immune cells, we then examined the association between MALT1 copy number and infiltration level of several immune cells in PCa, such as B cell, CD4^+^ T cell, CD8^+^ T cell, macrophage, neutrophil and dendritic cell (DC). The main function of B cell is to produce antibody mediated immune response and CD4^+^ T cells were reported to be helper T cells that are capable of promoting effective antitumor immune responses. CD8^+^ T cells mainly refer to cytotoxic T lymphocytes (CTLs), and an increase in CD8^+^ CTL levels could lead to efficient killing of tumors (Ness et al., 2014). In addition, macrophages can promote or inhibit tumor progression (such as cell proliferation, metastasis and invasion) (Gorchakov et al., 2020). Tumor-associated neutrophil cells (TAN) play an significant role in promoting angiogenesis, and they can affect tumor migration through releasing matrix metalloproteinase 9 (MMP9) (Granot, 2019). DC can activate B cells, NK cells and NK-T cells, induce and maintain tumor immune response (Garofano et al., 2019). In this study, we found that MALT1 expression was significantly correlated with the levels of these 6 types of infiltrating immune cells. Here, we also found that the deep deletion and high amplification of MALT1 significantly decreased the infiltration of CD8^+^ T cells and CD4^+^ T cells, respectively. These results indicate that MALT1 may lead to tumorigenesis or inhibit tumor progression by changing the TIL status in PCa. These novel findings constitute substantial progress in identifying the important role of MALT1 in immune infiltration.

The latest research demonstrated that MALT1 is indispensable for the development of regulatory T cells and plays a significant role in immune homeostasis (Gewies et al., 2014; Jaworski et al., 2014). In the current study, the expression of MALT1 was also related to the expression of some specific immune checkpoint genes across multiple tumors. Therefore, we speculated that MALT1 could impact cancer progression and metastasis by regulating the activity of immune checkpoint genes.

We also demonstrated that there is a relationship between MALT1 expression and TMB and MSI in some cancer types and that some tumors show coexpression of MALT1 and major MMR genes. MSI and high TMB (TMB-high) are promising pancancer biomarkers to guide immune checkpoint blockade (ICB) treatment. Previous studies have linked TMB and MSI to drug responses in patients, particularly drugs targeting immune checkpoint inhibitors, such as TGF-β antagonists and PD-1 inhibitors (Overman et al., 2017; Mariathasan et al., 2018; Kwon et al., 2020; Lagos et al., 2020; Shim et al., 2020; Yoshino et al., 2020). Therefore, we suggest that MALT1 expression be used as an additional indicator for the evaluation of immunotherapy in cancer patients after treatment. Interestingly, MSI is now considered an indicator of tumor types in patients with COAD. In addition, COAD patients with high MSI showed better checkpoint inhibitor response and survival in both early and late clinical stages (El Agy et al., 2019; Allan et al., 2020; Dzunic et al., 2020). In our study, both the TMB and MSI of COAD were positively correlated with the expression of MALT1, which supports our claim that MALT1 may be a good indicator of potential drug response (and MSI), as well as in COAD.

## Conclusion

This is the first time the cellular function of MALT1 in PCa was investigated, and its molecular mechanism was explored through bioinformatics analysis. Our findings confirm that MALT1 participates in promoting proliferation and colony genesis while reducing the level of apoptosis of PCa cells and is also predicted to be associated with migration and metastasis of PCa. Knocking down of MALT1 attenuated the tumorigenesis ability of PCa in mice. This study will add to our understanding of the molecular mechanism of PCa and hopefully provide novel targets for individualized cancer therapy. Furthermore, we identified Correlation of MALT1 expression with immune check points, TBM and MSI in most cancers by bioinformatic analysis. Authors have identified very significant correlation of MALT1 expression with tumor burden and various cancer driven pathways in multiple cancers. However, a limitation of our study is that only 1 cell line was studied *in vivo*. Thus, it is important to verify our findings in other NSCLC cell lines before reaching a final conclusion.

## Data Availability

The original contributions presented in the study are included in the article/Supplementary Material, further inquiries can be directed to the corresponding authors.
